# Assessing position changes of impacted third molars in treatment of class II malocclusion with premolars extraction

**DOI:** 10.2340/aos.v83.42076

**Published:** 2024-10-09

**Authors:** Hatice Gokalp, Murat Kaan Erdem

**Affiliations:** a Department of Orthodontics, Faculty of Dentistry, Ankara University, Ankara, Turkey; b Department of Oral and Maxillofacial Surgery, Faculty of Dentistry, Lokman Hekim University, Ankara, Turkey

**Keywords:** Class II malocclusion, moderate anchorage, orthodontic therapy with extraction, third molar impaction

## Abstract

**Introduction:**

Third molars (M_3_) remaining impacted in Class II malocclusion characterised with sagittal mandibular deficiency is a high probability. The null hypothesis of this study is that mesioangular M_3_s changes position through the eruption way in Class II malocclusion treatment requiring moderate anchorage with four first premolars extraction. The aim of this study is to reconsider the decision to surgically extract impacted third molars in four premolars extraction treatment of Class II malocclusion.

**Material and Methods:**

The materials consisted of the pre-treatment and post-treatment lateral cephalograms and orthopantomographs of 30 individuals with skeletal and dental Class II malocclusion with a mean chronological age of 13.48 years, who were treated by the same clinician (H.G.) with four first premolar extractions via the straight wire technique at the Ankara University Faculty of Dentistry Department of Orthodontics, Ankara, Turkey. The sagittal position of the upper and lower incisors and molars, M_3_ position and M_3_ space were evaluated with the paired-t test; the relationship between the sagittal position of the upper and lower incisors and molars and the change in M_3_ position were evaluated with correlation analysis.

**Results:**

The study found the retroclination and mesial movement of the upper incisors and molars, and an increase in the M_3_ space by the fixed orthodontic treatment. An insignificant steepening of both the upper right M_3_ position and the lower right M_3_ position was found. A statistically significant increase in the lower right and left side M_3_ spaces was found. Positive correlations between lower right M_3_ angulation and the sagittal position of the lower incisors and first molars were found.

**Conclusion:**

Improvement in the mesioangulation of the M_3_s and an increase in the M_3_ space were achieved in this study. Based on the findings, it is useful to review the decision for prophylactic surgical extraction of the M_3_s before orthodontic treatment in such cases, taking into account the risks of postoperative complications.

## Introduction

Tooth impaction is a common occurrence, affecting 0.8% – 3.6% of the general population, mainly because of space constraints in the dental arches or other conditions that hinder eruption. These are influenced by systemic, local, genetic, and racial factors [1, 2]. The teeth most frequently impacted include maxillary and mandibular third molars, maxillary canines, mandibular premolars, and maxillary central incisors [3, 4].

Mandibular third molar (M_3_) impaction was initially attributed to inadequate space between the second molars (M_2_) and the ascending ramus. Later studies identified additional contributing factors, such as mandibular growth deficiency, vertical condylar growth, and the backward eruption path of the dentition [5, 6].

In recent times, changes in eating habits that affect chewing patterns, coupled with insufficient jaw lengthening, have resulted in an increased prevalence of M_3_ impaction, ranging from 16.7% to 68.6% [7]. Fossil records indicate a reduction in both the number and size of individual teeth and jaw size throughout evolution. Initially, primates had more teeth, but over time, the third premolars and fourth molars disappeared in mammals. Presently, it is not uncommon for M_3_s, second premolars, and lateral incisors to sometimes fail to form [8].

Orthodontic treatment often requires the extraction of first premolars to achieve desired treatment outcomes. However, in treatments characterised by a forward mandibular growth pattern, the length of the dental arch may decrease, leading to the impaction of M_3_s because of factors such as ramus anterior remodelling, late mandibular growth spurt, and retrusion of the lower incisors [1, 6, 8–12]. In orthodontic treatment plans, regardless of whether extractions are involved, the common approach is to prophylactically extract asymptomatic M_3_s, independent of craniofacial growth characteristics. However, the margin of error in estimating the risk of impaction is 40% [13, 14]. This substantial uncertainty is particularly concerning given that surgically extracting asymptomatic M_3_s in adolescents can lead to long-lasting neurological and psychological complications [15, 16]. Therefore, it is crucial to evaluate the prognosis of M_3_ eruption alongside anchorage requirements, craniofacial growth patterns, M_3_ angulation, and M_3_ spacing to minimise surgical complications.

In cases of Class II malocclusion requiring maximum anchorage, distal molar movement may increase the risk of M_3_ impaction because of the influence of craniofacial growth direction on the remodelling of the ascending ramus [6].

Numerous studies on M_3_ impaction in the treatment of Class I, II, and III malocclusions, with or without extraction and during or after the growth period, are retrospective. These studies often fail to prioritise the relationship between anchorage requirements and craniofacial growth characteristics in M_3_ impaction. However, the craniofacial growth pattern significantly influences tooth alignment within the alveolus.

The null hypothesis of this study is that mesioangular M_3_s undergo positional changes during the eruption process in the course of treating Class II malocclusion, particularly in cases that require moderate anchorage and involve the extraction of all four first premolars. The primary aim of this study is to critically reevaluate the decision-making process regarding the surgical extraction of impacted third molars within the framework of Class II malocclusion treatments that include the extraction of four premolars.

## Materials and methods

### Sample design

This study was conducted on the lateral cephalograms and orthopantomograms (OPG) of 30 patients with skeletal and dental Class II malocclusion requiring moderate anchorage. These patients were treated by the same orthodontist (H.G.) using the straight wire technique with four premolar extractions at the Ankara University Faculty of Dentistry, Department of Orthodontics. All premolars were extracted by the same surgeon (M.K.E.). A power analysis determined that a sample size of 30 was adequate. All radiographs were taken by the same technician using a Planmeca ProMax Device, set at 66 kV and 9 mA, with the patients’ mouths closed. The mean treatment duration was 2.7 years (range: 1.90–4.50 years).

**Figure 1:**
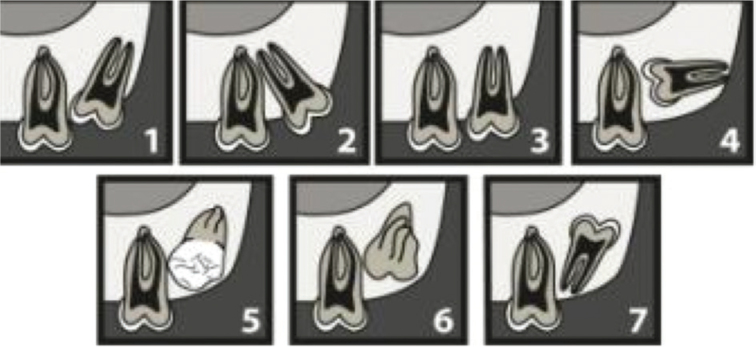
Archer’s classification of upper third molars according to their inclination to the long axis of the upper second molar. (1) mesioangular, (2) distoangular, (3) vertical, (4) horizontal, (5) buccoangular, (6) linguoangular, (7) inverted.

**Figure 2:**
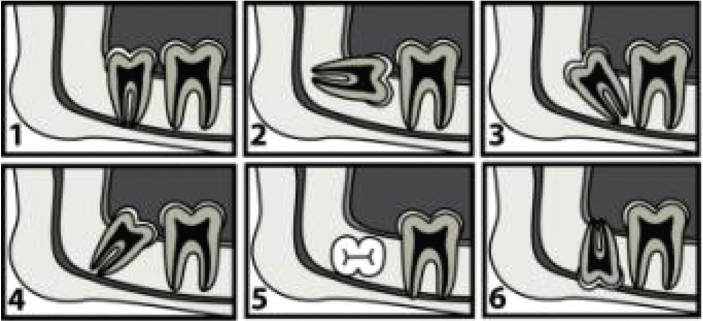
Winter’s classification (19): Third molars are classified according to their inclination to the long axis of the second molar. (1) vertical angulation, (2) horizontal angulation, (3) distoangular angulation, (4) mesioangular angulation, (5) transversal angulation, (6) inverse angulation.

The average chronological age of the individuals at the beginning of treatment was 13.48 years (range: 11.80–19.30 years). Inclusion criteria were:

1. Maxillary and mandibular arch length discrepancy with moderate anchorage requirements: −7.45 mm and −5.19 mm, respectively; Class II molar and canine relationship; overjet: 4.90 mm (range: 1.00–15.00 mm); overbite: 2.15 mm (range: −4.0 to 5.0 mm).
2. SNA, 85º; SNB, 79º; ANB, 6º.
3. GoGn-SN, 35.4º.
4. All third molars present and in a mesioangular position on OPG, according to Archer’s and Winter’s classifications ([17, 18]; Figures 1 and 2).

**Figure 3:**
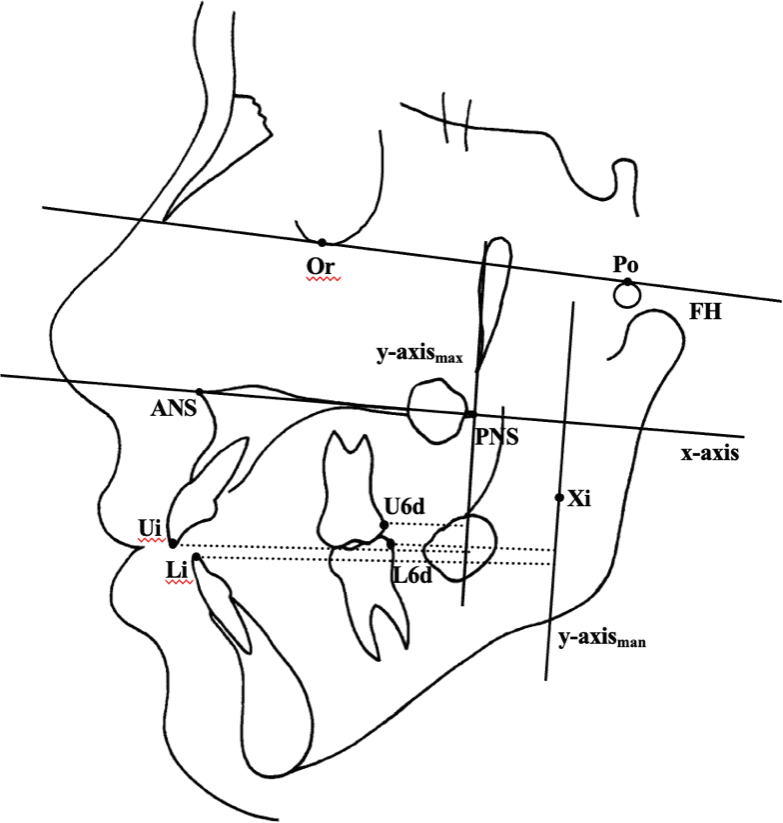
Points and reference lines for measurement of Lateral cephalograms. **Points.** 1. ANS, anterior nasal spine; 2. PNS, posterior nasal spine; 3. Ricketts Xi point 4. Ui, upper incisor edge, 5. U6d, upper first molar distal edge, 6. Li, lower incisor edge, 7. L6d, lower first molar distal edge. **Reference Lines:** 1. FH, Frankfort horizontal line; 2. X-axis is made between the ANS and PNS points. 3. Y-axis for maxilla is perpendicular line from PNS point to x-axis. 4. Y-axis for mandible is perpendicular line from Xi point to x-axis. Measurements: 1**.** Ui- y-axis_max_,2. U6d- y-axis_max_, 3. L6d- y-axis_max_ 4. L6d- y-axis_man_. 5. M_3_ space for maxilla: distance between U6d and y-axis_max._ 6. Distance between L6d and y-axis_man_.

**Figure 4:**
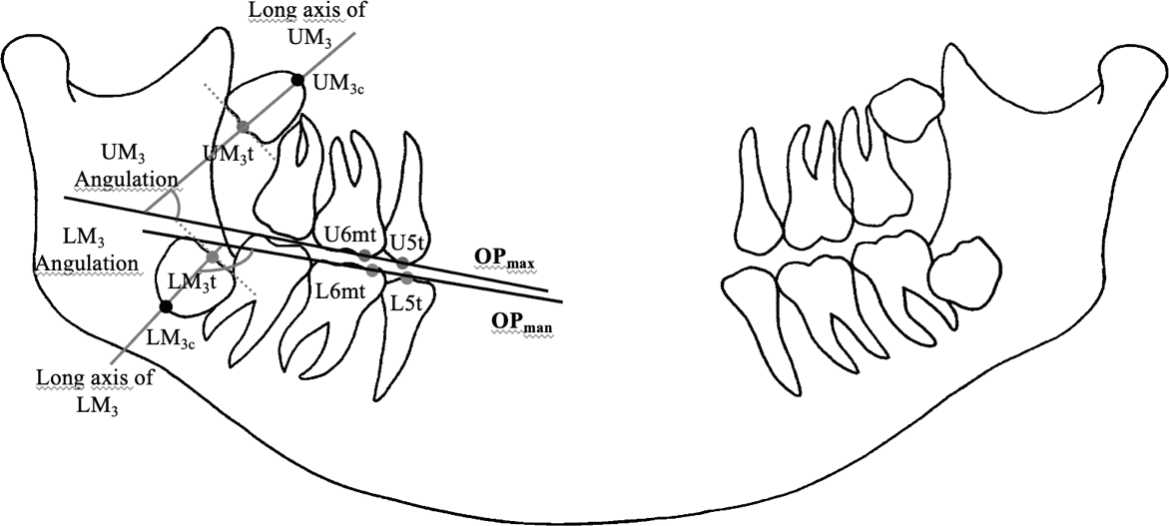
Points and reference lines for measurement of OPG. **Points:** 1. UM_3_t, Upper third molar occlusal surface midpoint 2. U6mt, Upper first molar mesial tubercule top 3. UM_3c_ Upper first molar midpoint of apex. 4. U5t, upper second premolar tubercule top, 5. LM_3_t, lower third molar occlusal surface midpoint 6. L6mt, lower first molar mesial tubercule top 8. LM_3c_ lower first molar midpoint of apex_._ 9. L5t, lower second premolar tubercule top. **Reference Lines:** 1. OP_max_,maxillary occlusal plan which is constracted between U5t and U6mt. 2. OP_man_, mandibular occlusal plan which constracted between L5t and L6mt, 3. Long axis of UM_3,_ line between UM_3_c and UM_3_t. 4. Long axis of LM_3,_ line between LM_3_c and LM_3_t. Measurements: 1. UM_3_ angulation, angle between OP max and long axis of UM_3._ 2.LM_3_ angulation, angle between OP man and long axis of LM_3_.

At the end of orthodontic treatment, occlusion was achieved in accordance with Andrews’ normal occlusion criteria [19]. On OPG, all M_3_s were present, and at least one-third of root formation was completed. Temporary intraoral anchorage systems or extraoral anchorage applications were not used during fixed orthodontic treatment. Changes in M_3_ space and angulation were evaluated on lateral cephalograms and OPGs at the end of the treatment (Figures 3 and 4).

### Study design

To evaluate sagittal changes in the positions of incisors and molars and M_3_ spaces in both dental arches, a Cartesian coordinate system was used. The X-axis was created between the anterior nasal spine (ANS) and posterior nasal spine (PNS) points, and was used for both upper and lower dental arches. The Y-axis for the maxilla was constructed from the PNS point to the X-axis. Sagittal changes in the positions of the upper incisors, molars, and M_3_ spaces were measured relative to the Y-axis. The Y-axis for the mandible was constructed from the point where the anterior border of the ascending ramus joins the end of the corpus mandible to the X-axis. Sagittal changes in the positions of the lower incisors, molars, and M3 spaces were measured relative to the Y-axis. M_3_ spaces for upper and lower dental arches were measured as the distances between the U6d and Y-axis (maxilla), and L6d and Y-axis (mandible). Reference landmarks and lines for measurement on lateral cephalograms are presented in Figure 3. Positional changes of the M_3_s were evaluated on OPG. Points and reference lines used on OPG are shown in Figure 4.

### Statistical method

SPSS (Statistical Package for the Social Sciences) 26 was used for data analysis. The intraclass correlation coefficient was used to measure reliability. Because of the limited data for comparing the beginning and end of the treatment using lateral cephalogram and OPG measurements (*N* = 30), the nonparametric Wilcoxon signed-rank test was used as an alternative to the dependent group t-test for comparing two different measurements within a single group [20]. The nonparametric Brown correlation method was used instead of the Pearson correlation method to assess measurement differences between the beginning (T0) and end (T1) of treatment. The correlation coefficient (*r*) was considered low if below 0.40, medium if between 0.40 and 0.70, and high if equal to or greater than 0.70 [21]. A significance level of *p* < 0.05 was used for statistical analyses.

Measurements were conducted twice with a 20-day interval to determine the repeatability of landmark identification and measurement techniques. All angular and linear variables exhibited a coefficient of intra-rater reliability between 0.82 and 1.00, indicating negligible variation.

Sagittal position changes of the incisors and molars, M_3_ spaces, and mesioangular M_3_s were analysed using a paired-t test at T1. The relationship between changes in the positions of incisors/first molars and changes in M_3_ positions and M_3_ spaces were tested using correlation analysis.

## Results

At the end of orthodontic treatment, a statistically significant retraction of the upper incisors and mesialization of the molars was observed (*p* < 0.01), along with a significant increase in the upper M_3_ space (*p* < 0.01, Table 1). Although there was no change in the lower incisor position, a statistically significant mesialization of the lower molars and an increase in the lower M_3_ space were found (*p* < 0.01, Table 1).

**Table 1:** Changes of upper and lower incisors and first molars in addition to M_3_ spaces in both arches at T0 and T1.

*n* = 30	Before Treatment (T0)	After Treatment (T1)	*p*
	X ± Sx	X ± Sx	
U1 position (mm)	51.52 ± 4.22	48.68 ± 4.17	**
U6 position (mm)	22.42 ± 4.12	24.95 ± 3.42	**
M_3 max_ space (mm)	10.13 ± 3.18	12.65 ± 3. 55	**
L1 position (mm)	50.68 ± 3.47	50.25 ± 4.61	Ns
L6 position (mm)	27.37 ± 3.15	30.23 ± 3.79	**
M_3 man_ space (mm)	13.20 ± 3.17	16.06 ± 6. 53	**

Significance level: Ns: Not significant;

** *p* < 0.01.

A statistically significant decrease in the right lower M_3_ angulation was detected at the end of the treatment (*p* < 0.05, Table 2), while no significant changes were observed in the positions of other M_3_s.

**Table 2:** Sagittal position changes of M_3_s on OPG by orthodontic treatment.

	Before Treatment (T0)	After treatment (T1)	*p*
	x ± S×	x ± S×	
Long axis of URM_3_/OP_max_	63.04 ± 20.49	65.54 ± 11.42	Ns
Long axis of LRM_3_/OP_man_	146.72 ± 18.32	141.10 ± 21.14	*
Long axis of ULM_3_/OP_max_	61.52 ± 18.31	64.48 ± 15.10	Ns
Long axis of LLM_3_/OP_man_	134.88 ± 19.05	131.52 ± 21.11	Ns

Significance level: Ns: Not significant;

* *p* < 0.05, **p < 0.01.

A statistically significant positive correlation was found between treatment and changes in the positions of U6 and L6 (*p* < 0.05, Table 3). Additionally, a statistically significant positive correlation was found between the lower right M_3_ position and the positions of the lower incisors and lower first molars (*p* < 0.01, Table 3).

**Table 3:** Correlations analysis results between changes of sagittal positions of the upper and lower incisors and molars and M_3_s position changes.

	U6	L1	L6
L6	0.417*	Ns	Ns
Long axis of LRM_3_/OP_man_	Ns	0.480**	0.484**

Significance level:

* *r*_0.05_ = 0.374,

** *r*_0.01_ = 0.479.

## Discussion

This study examined the changes in the required space for the eruption of mesioangular M_3_s and their angulation during fixed orthodontic treatment involving four premolar extractions and moderate anchorage requirements. While many studies have addressed this topic, controversies remain regarding craniofacial growth patterns, impaction detection methods, and orthodontic treatment planning. This study found that fixed orthodontic treatment with four premolar extractions, requiring moderate anchorage, led to upper incisor retraction, molar mesialization, an increase in the space necessary for M_3_ eruption in both the upper and lower dental arches, and an improvement in the mesioangular M_3_ position.

Anchorage requirements play a critical role in tooth movement during orthodontic treatments involving extractions. Different anchorage systems yield varying results in tooth positioning. In this study, patients were treated only with fixed orthodontic treatment, without the use of intraoral or extraoral anchorage systems. Increased anchorage requirements in the orthodontic treatment of Class II malocclusion, necessary for retracting the upper incisors and molars, may contribute to M_3_ impaction.

Orthopantomograms were utilised to assess M_3_ angulation in this study. Although cone-beam computed tomography (CBCT) has gained popularity, OPGs remain a standard practice because of their routine use and lower radiation doses in orthodontics [22, 23].

Various factors influence the space available for M_3_ eruption. Richardson noted that mesial molar movement can partially increase the space for M_3_ eruption [1]. Brash and Scott observed that anterior dentition movement contributes to creating space for M_3_ eruption [24, 25]. Premolar extraction has been found to increase the space required for M_3_ eruption [26]. Ricketts reported that in premolar extraction treatment, the space required for mandibular M_3_ eruption increases by 25%, necessitating early M_3_ prognosis evaluation [27]. In non-extraction orthodontic treatment, 45% of M_3_s must be extracted, compared to 15% – 20% in treatments involving first premolar extractions.

This study found that orthodontic treatment with premolar extractions positively influenced the mesioangular position of M_3_s. Moderate anchorage needs and skeletal growth were important factors. Conversely, some studies suggest that growth has little effect on changes in M_3_ angulation, and orthodontic treatment at the end of the growth period may not significantly affect M_3_ angulation [28, 29]. Although literature indicates that extraction-inclusive treatments positively impact the necessary space for M_3_ eruption, particularly in the lower jaw, factors such as growth model, treatment technique, and anchorage needs have not been fully considered [1, 9, 10, 30].

Orthodontic treatment with premolar extraction, requiring moderate anchorage, contributed positively to increasing the distance between the ascending ramus and the distal surface of the M_2_s, facilitating the eruption of M_3_s in a forward skeletal growth pattern. Based on these results, the decision for surgical extraction should be carefully evaluated, considering the potential risk of psychological trauma associated with the surgical extraction of third molars during adolescence, either before or after orthodontic treatment.
